# Supplementary material for: Are carbon emissions trading and green financial instruments synergistic? -Comprehensive quantitative research based on content analysis
Source: PLoS One. 2024 Mar 7;19(3):e0298601. doi: 10.1371/journal.pone.0298601 (PMC10919601; doi:10.1371/journal.pone.0298601)
Supplement: S1 Data — (ZIP) [file pone.0298601.s001.zip › Data/地级市Z_F工作报告.zip!/║╙─╧/┐¬╖Γ╩╨/└┤╫╘╣1⁄2╓┌║┼í╛┬φ┐╦╩2╛▌═°í┐_2013.docx]

各位代表：

现在，我代表市人民政府向大会作工作报告，请予审议，并请各位政协委员和列席人员提出意见。

一、2012年工作回顾

刚刚过去的2012年，面对复杂严峻的经济形势，全市上下在省委、省政府和市委的正确领导下，在市人大常委会、市政协的监督支持下，深入贯彻落实科学发展观，牢牢把握“持续求快”总基调，全力打好 “五大攻坚战”，科学谋划，积极作为，务实发展，较好地完成了市十三届人大五次会议确定的各项任务。全市生产总值完成1212.2亿元，增长11.1%；规模以上工业增加值390.1亿元，增长17.8%；固定资产投资738.2亿元，增长26.1%；公共财政预算收入61.9亿元，增长26.2%，其中市本级26.8亿元，增长18.2%；社会消费品零售总额503.5亿元，增长16%；城镇居民人均可支配收入17545元，增长12.8%；农民人均纯收入7414元，增长14.2%。固定资产投资、公共财政预算收入增幅均居全省第1位，规模以上工业增加值、社会消费品零售总额增幅分别居全省第3位和第5位，主要经济指标增幅继续位居全省前列。

（一）工业经济持续向好。深入开展企业服务活动，全市规模以上工业企业达到1121家，完成主营业务收入1602.5亿元，增长16.2%，主营业务收入超10亿元企业达到23家，工业用电量增长10.8%。重点项目实现新突破，全市315个重点项目完成投资564.8亿元，增长27%。全部工业投资完成442亿元，增长28.6%。晋开百万吨总氨、奇瑞汽车河南生产基地等重大项目建成投产。产业集聚区建设明显加快，8个产业集聚区完成固定资产投资470.4亿元，增长32.9%。产业集群培育成效明显，十大产业集群规模以上工业企业完成主营业务收入1331亿元，增长14.5%。

（二）新区建设及郑汴一体化持续推进。新区基础设施配套不断完善，13条道路建成通车，金明大道北延、金明广场综合整治工程竣工，开封西湖工程加快推进，中心商务区启动建设，职业教育园区入住学生1万人。迁村并居工程扎实推进，安置房封顶53.9万平方米。主导产业培育力度加大，奇瑞重工、住成电装等项目建成投产，新区入驻企业达到227家，汽车及零部件、现代装备制造等主导产业初具规模。金明区平稳并入开封新区，管理体制进一步理顺。郑汴一体化取得新进展，郑汴物流通道建成通车，郑开城际铁路、连霍高速开封段扩宽等工程扎实推进。金融同城顺利实现，电信同城正在加紧对接，尉氏县部分区域纳入郑州航空港经济综合实验区规划。

（三）文化旅游等服务业持续发展。成功举办2012清明文化节、中国（开封）第30届菊花文化节、中国收藏文化（开封）论坛、第22届中国厨师节，开封被命名为全国首座“中国收藏文化名城”，全市旅游接待量和旅游综合收入分别增长13%和16%。鼓楼复建、城墙保护五期等一批文化旅游项目进展顺利，金融、商贸、邮政、电信、交通运输和房地产业健康发展，开元商业广场建成开业，交通银行在开封设立分支机构。全市金融机构新增贷款110亿元，增长24.9%，增幅居全省第1位。全市第三产业增加值416亿元，增长11%，增幅居全省第3位。

（四）城乡面貌持续改观。老城区改造力度加大、步伐加快，大规模实施“两改一建”工程，完成棚户区、城中村改造面积258.7万平方米，新开工建设保障性住房12307套。不断完善城市基础设施，内顺城路拓宽改造（迎宾门-向阳路）、河南大学周边环境整治、东护城河景观整治（一期）等项目顺利完工。数字化城市管理系统运行水平不断提高，依法整治规范市区三轮车成果进一步巩固，启动整治规范犬类工作，深入开展市容秩序、交通秩序和环境卫生集中整治工作，城市品位持续提升。县城和中心镇建设明显加快，编制完成五县县域村镇体系规划、五区村庄布局规划，扎实开展“双改”工作，启动实施县城建设“三年百楼”和“十个一”工程。新型农村社区建设扎实推进，开工建设新型农村社区104个，累计建房2.6万套，30个社区初步形成规模。

（五）农业基础持续巩固。落实各类强农惠农富农资金8.6亿元。全市粮食总产267万吨，增长3.16%，实现“九连增”。肉、蛋、奶总产量分别达到38.5万吨、25.7万吨、26.6万吨，增长4.6%、3.6%、4.4%。优质农作物种植面积超过60%，主要农作物良种覆盖率99%。省级以上重点农业产业化龙头企业增加到20家，新发展农民专业合作社528家、农机专业合作社36家，农业综合机械化率达到79.5%。投资1.9亿元，完成48个贫困村整村推进建设任务，6.2万农村人口实现脱贫。

（六）对外开放持续扩大。大力开展招商引资，全市新签约亿元以上项目177个，新开工亿元以上项目174个。引进省外资金376.8亿元，增长44%。实际利用外资3.6亿美元，增长55%。境外合作实现新突破，新获得进出口经营权企业50家，完成出口额2.9亿美元，增长26%。发展环境不断优化，我市再次当选“中国最具投资潜力城市50强”。

（七）发展方式持续转变。高新技术企业达到14家，省级以上工程技术研究中心增加到12家，柔性引进两院院士8人。强化主要污染物排放总量控制，完成PM2.5监测设备安装和运行工作，全年空气环境质量优良天数达到300天，饮用水源地水质达标率保持100%。全面完成省定淘汰落后产能任务，单位生产总值能耗较上年下降6%。加强土地资源管理调控，连续13年实现耕地占补平衡。

黄河滩区砖瓦窑厂全部关闭，复耕土地1.8万亩。抓好林业生态建设，造林12.6万亩。

（八）民生得到持续改善。坚持公共财政向民生倾斜，全市投入民生资金118.5亿元，占公共财政预算支出的69%。加大就业创业支持力度，强化就业技能培训、创业培训，发放小额担保贷款8.8亿元，城镇新增就业8万人，其中下岗失业人员再就业3.2万人，城镇登记失业率3.66%。社会保障水平不断提高，城市和农村低保标准分别提高到年人均3120元和1500元，农村五保年分散供养、集中供养标准进一步提高。医疗、工伤、生育、失业保险实现市级统筹，城乡居民社会养老保险实现全覆盖。企业养老保险费征缴16.9亿元，失业保险费征缴突破1亿元。新农合参合率99.8%，人均筹资水平提高到290元，补偿资金11.5亿元。大力推进义务教育均衡发展，全市教育支出33.5亿元，新改扩建幼儿园150所，开封大学新校区建设加快推进。完成市人民体育场（馆）改建工程，成功举办2012中国郑开国际马拉松赛。公共文化服务能力不断提升，开展“舞台艺术送农民”演出181场，为农民放映公益电影2.9万场，“农家书屋”实现行政村全覆盖。深化基层医疗机构综合改革，基层医疗卫生单位实施基本药物制度。稳定低生育水平，人口自然增长率6.1‰。优先发展公共交通，更新城市公交车100辆，新增市区出租车300辆。提高社会管理科学化水平，健全食品药品安全综合协调和监督机制，强化安全生产监管，严厉打击各种违法犯罪。进一步加强信访稳定工作，健全矛盾纠纷排查调处机制，化解了一大批信访积案，社会大局保持和谐稳定。

（九）政府自身建设和民主法制工作持续加强。深入开展“学整树”活动，严格落实效能问责办法，加快行政审批制度改革，各级政府及各部门服务效能和工作水平进一步提升。坚持依法行政，自觉接受人大依法监督、政协民主监督和社会监督，认真办理省市人大议案、代表建议和政协提案1080件，满意率97.5%。贯彻落实“六五”普法规划，依法治市工作取得明显成效。扎实推进政府廉政建设，强化行政监察和审计监督。大力开展国防教育，扎实做好“双拥”工作，驻汴解放军和武警部队在支持开封经济建设、维护社会稳定等方面作出了重要贡献。民族、宗教、统计、外事、侨务、对台、档案、史志、地震、人防、气象、社科研究等工作都取得了新的成绩。

各位代表！

过去的一年，我们谋长远、抓大事、解难题、惠民生，取得了令人振奋的成绩，这是在经济形势复杂严峻、外部竞争十分激烈的情况下，抢抓机遇、科学谋划、有效运作的结果；是在自身发展基础薄弱、要素制约加剧的环境中，转变作风、克难攻坚、务实发展的结果。这些成绩的取得，得益于省委、省政府和市委的正确领导，得益于市人大、市政协的监督支持，凝聚着全市人民的智慧和汗水。在此，我代表市人民政府，向全市工人、农民、知识分子、干部，向驻汴解放军指战员、武警官兵和公安民警，向各民主党派、工商联及社会各界人士致以崇高的敬意！向所有关心、支持开封发展的同志们、朋友们表示衷心的感谢！

在总结成绩的同时，我们也清醒地认识到，我市底子薄、基础弱、人均水平低的基本市情尚未根本改变，发展中还有许多困难和问题。主要表现在：经济总量依然偏小，生产总值总量居全省第13位，公共财政预算收入总量居全省第11位，固定资产投资总量居全省第15位；三次产业结构不合理，第二产业实力偏弱，二、三产业比重低于全省平均水平8.5个百分点；城镇化水平低，县城和小城镇规模偏小，基础设施欠账较多；城乡居民收入水平总体不高，一些涉及群众切身利益的问题尚未得到很好解决；资金、土地、资源环境等制约因素日益突出，经济社会可持续发展的瓶颈亟待破解；政府职能转变还有差距，一些政府工作人员全局意识、服务意识、责任意识、为民意识还不够强，工作方法、工作作风、能力素质与新形势、新任务的要求不相适应。对此，我们一定高度重视，采取有效措施，认真加以解决。

2013年是全面贯彻落实党的十八大精神的开局之年，是实施“十二五”规划的关键一年，也是实现开封快速崛起、为全面建成小康社会奠定坚实基础的重要一年。新的一年，我们肩负着加快发展、加速转型、改善民生等多重任务，既要保持较快的经济发展速度，尽快缩小与先进地区的差距，又要真正转变发展方式，不断提高发展的质量和效益，更要解决好涉及人民群众切身利益的就业、就学、就医、住房、环保、食品安全等民生问题。面对新形势新任务新要求，全市上下人心思进、干事创业的浓厚氛围已经形成，抢抓机遇、加快崛起的态势已经形成，各县区竞相发展、晋位升级的强大合力已经形成，总体上看，我市仍处于风生水起、可以大有作为的战略机遇期。只要我们切实增强历史责任感和现实紧迫感，抓住用好机遇，奋力开拓创新，就一定能在实现开封快速崛起的道路上迈出更加坚实的步伐。

2013年政府工作的指导思想是：深入学习和全面贯彻党的十八大精神，以邓小平理论、“三个代表”重要思想、科学发展观为指导，坚持“四个重在”实践要领，围绕“持续求快”总基调，抢抓中原经济区战略实施、郑汴一体化发展、郑州航空港经济综合实验区建设重大机遇，持续推进“五大攻坚战”，着力增加经济总量，着力培育主导产业，着力提升城镇形象，着力保障和改善民生，努力建设实力开封、文化开封、美丽开封、幸福开封。

2013年全市经济社会发展的主要预期目标是：生产总值增长11%；公共财政预算收入增长15%；规模以上工业增加值增长18%；固定资产投资增长23%；社会消费品零售总额增长16%；外贸进出口总额增长15%；实际利用外资增长20%；城镇居民人均可支配收入和农民人均纯收入增长11%；城镇新增就业5.8万人以上；人口自然增长率控制在6.5‰以内；城镇登记失业率控制在4.5%以内；居民消费价格涨幅3.5%左右；节能减排完成省下达任务。

实现上述目标，要重点抓好以下10个方面的工作：

（一）做大做强工业。坚定工业强市不动摇，以8个产业集聚区建设为载体，以十大产业集群培育为重点，加快新型工业化步伐。大力培育主导产业骨干企业。全面落实《十大产业集群“十二五”发展规划》和《加快培育发展十大产业集群的意见》，有针对性地开展招商引资，承接产业转移，力争晋开集团主营业务收入达到80亿元，用3~5年时间，培育一批主营业务收入超500亿元的产业集群、主营业务收入50亿元以上的骨干企业。加快工业企业科技创新，新建6家省市工程技术研究中心，实施5个以上高新技术产业化项目。大力实施质量立市和品牌带动战略，新培育国家级名牌产品两个、省级名牌产品6个。完善现代企业制度，加快推进优势企业上市。狠抓工业项目建设。把项目建设作为打好工业强市攻坚战的重要突破口，全力推进河南煤化集团科技研发中心及制造基地、龙宇化工三期、炭素二期扩建、通许电动车产业园等10个重大工业项目实施，确保完成工业投资450亿元以上。着力加强企业服务。尽快出台减轻企业负担、促进产销对接、金融支持等服务措施，积极帮助企业抓生产促发展、挖潜能保市场、想办法解难题，把事关企业管理经营以外的事情办实办好。更加重视民营经济及中小微型企业发展，建立健全政策扶持、人才支撑体系，全力为企业营造良好发展环境。加快产业集聚区建设与发展。继续推进“四集一转”，做强做大主导产业，拉长产业链条，力争8个产业集聚区主营业务收入达到1200亿元，增长20%以上。加快信息化建设。继续实施“两化”融合试验区示范工程，采用先进信息技术改造提升传统产业，创建一批省“两化”融合示范企业。提升城市信息系统综合应用水平，紧紧围绕城市建设管理、旅游产业发展、民生需求等，建成一批城市数字化应用系统，努力建设“智慧开封”。

（二）加快开封新区建设。今年是开封新区五年成规模的关键一年，要以产业作支撑，以城建出形象，保持好势头，实现新跨越。加快开发建设，打造新区样板。开拓多元化融资渠道，抓好开封新区启动区、铁南工业区、北大资源运粮河组团以及郑开大桥、东京大桥、晋安大桥、新区污水处理厂等基础设施建设，确保基础设施投资完成40亿元。完善道路、绿化、供排水、供电、供气等设施配套，完成开封西湖土方开挖任务，提高新区吸纳承载能力。把“八园”（德豪光电科技园、中原生物产业园、中原家居产业园、富士康科技园、北大科技园河南园区、河南银基文化产业园、汽车零部件产业园、职业教育园区）、“两馆”（规划馆、博物馆）、“一湖”（开封西湖）、“一中心”（中心商务区），作为新区项目建设的重点，建成一批彰显新区风采、展示开封形象的现代城市综合体。加快迁村并居步伐，保障重大项目顺利实施。培育特色产业，打造发展引擎。把做强特色主导产业作为新区发展的主线，支持新区在土地、规划、投融资、产业发展等领域积极探索，进一步壮大汽车及零部件、空分设备、煤机专用设备三大制造基地规模，积极培育电子信息、新材料、新能源和生物医药等新兴产业，力争新区规模以上企业主营业务收入突破200亿元。

（三）推进新型城镇化发展。坚持新型城镇化为引领，按照中心城区、县城、中心镇、新型农村社区“四位一体”发展模式，构建以中心城区为核心、县城为支撑、20个中心镇为支点、800个新型农村社区为依托的新型城镇化格局。继续大力推进“两改一建”。坚定信心，着力民生，加快进度，破解融资、征收、安置房建设等难题，完成中心城区棚户区改造180万平方米，城中村改造170万平方米，五县完成房屋征收350万平方米。加快县城和小城镇建设步伐。完善县域经济扶持激励考核机制，大力发展县域经济，提高县域综合实力，实现更高层次的晋位升级。深入实施城乡建设三年大提升计划，加快县城建设步伐，壮大县城规模，抓好朱仙镇等中心镇开发建设，加快城乡交通基础设施建设，实施省道326杞县境，省道102、省道220尉氏境等干线公路升级改造工程。推进新型农村社区建设。坚持新型农村社区建设向县城和县城周边集中、向产业集聚区集中、向中心镇集中，统筹好政府引导与群众自愿、开发投资者利益与群众利益、住宅建设与配套设施建设、社区建设与产业发展的关系，解决好土地复耕、社区管理等问题，完善规划，优化布局，强力推进，着力改善农村面貌，提高农民生活质量。

（四）建设国际文化旅游名城。充分发挥文化旅游资源优势，突出文商旅结合，以“三城联创”为载体，着力建设国际文化旅游名城，不断探索并逐步形成“外在古典、内在时尚”的新宋风城市风格。以宋都古城文化产业园区建设为重点，加快文化旅游产业发展。推进“一城二环八区”建设，重点抓好清明上河城一期、水系二期周边、中原明珠文化旅游产业园、朱仙镇国家文化生态旅游示范区、城墙五期及城墙展示馆、七盛角民俗文化区、体育中心建设项目。持续提升中国（开封）菊花文化节、清明文化节、中国收藏文化（开封）论坛等节会水平，高标准筹备好第27届世界客属恳亲大会。提升《大宋·东京梦华》实景演出水平，运作推出室内文化旅游演艺剧目《一城宋韵》。积极发展休闲、健身、生态等深度旅游，启动黄河生态游览区开发建设。抓好游客集散中心、购物中心、星级酒店、旅游咨询点等旅游配套服务设施建设，创建省级旅游标准化示范城市，构筑符合国际文化旅游名城的硬件基础、物质形象和功能内涵，确保旅游接待量和综合收入实现两位数增长。以现代服务业为引领，大力发展第三产业。开展“知名品牌进开封”活动，吸引一批国际国内知名品牌进驻，提升商业服务层次。以老字号、老品牌为依托，运用现代技术、经营理念和管理手段，改造提升传统服务业，打造名吃、名店、名品和特色商业街区。围绕仓储物流、金融保险、信息技术、商务服务、科技研发五个方面，大力发展生产性服务业。以“三城联创”为载体，不断提升城市品位。加快复兴大道、劳动路北延、电视塔南路西路、内顺城路（向阳路—西门大街）、夷山大街南延、迎宾路北延（省府西街—西门大街）等城市道路建设步伐，科学规划建设停车场所，完善城市服务配套设施。着眼长远，统筹东区、北区、南区规划建设。提升数字化城市管理水平，深入实施犬类整治规范工作，推进城乡环境卫生清洁工程，整治交通秩序，进一步提高市民文明素质和城市文明程度。

（五）提高农业现代化水平。认真落实强农惠农富农政策，持续加大农业投入，大力发展现代农业。实施高标准粮田“百千万”建设工程。持续推进粮食核心区建设，抓好47个高产万亩方和尉氏、通许15万亩高标准粮 田建设，力争粮食总产量55亿斤以上，实现粮食“十连丰”。大力发展现代农业。全面推动农业科技创新，加快推进中国农业大学开封实验站建设，促进农业科技成果转化和推广，推动小麦、玉米、水稻、大蒜等主导作物品种换代升级。围绕提高农业综合效益，培育发展规模化、标准化、专业化农产品生产加工基地，加快皓月肉牛、雏鹰生猪、大用肉鸡、海壹食品等农业产业化龙头企业发展。抓好国际农业会展中心、宏安集团农产品物流、精准农业智能装备试验示范基地、7000亩菊花高新科技产业园及10个年度重点农产品批发市场等项目建设。抓好水利基础设施建设。加强以抗旱除涝为重点的农田水利工程建设和中小河流治理，完成三义寨、赵口、柳园口三个大型灌区续建配套、节水改造和黑岗口中型灌区建设任务，新增改善有效灌溉面积32.3万亩，解决30万农村居民饮水安全问题。创新农业生产经营体制机制。鼓励引导农民土地依法自愿流转，着力培育一批专业合作经济组织和新型经营主体，提高农业组织化程度。

（六）加快郑汴一体化发展。郑汴一体化是推动开封快速崛起的重大战略机遇，要积极主动借力省会、对接郑州，推动金融同城、电信同城、交通同城、产业同城、生态同城和资源共享向深层次发展。做好金融同城后续服务工作，争取更多金融、保险、证券机构在开封设立分支机构。配合完成固定电话升位并网、统一区号，实现移动和固定电话按市话收费。着力推进总投资230亿元的“四条高速”（连霍高速开封段拓宽、商登高速开封段、郑民高速二期杞县段、机西高速尉氏段）、“三条铁路”（郑开城际铁路开封段、郑徐客运专线开封段、新开铁路改扩建开封段）、“一个出口”（连霍高速开封新区新出口）建设。积极融入郑州航空港经济综合实验区，做好尉氏区域空间规划、产业规划，明确发展定位，努力打造开封经济发展新的增长板块。

（七）抓好重点项目建设。坚持把抓项目、扩投资作为保持经济“持续求快”的基本抓手，以大思路谋划大项目，以大项目引领大投资，以大投资促进大发展。认真研究国家投资重点，围绕国家确定的投资领域，抓好重大项目包装、论证，做好政策、项目、资金对接落实，着力在“五大攻坚战”、产业集聚区、城区“两改一建”、民生等领域谋划一批新的重大项目，积蓄发展后劲。组织实施300个以上重点项目，突出“五大攻坚战”5大类50个重大项目，发挥示范、引领、带动作用，确保当年完成投资600亿元。继续实施市级领导分包重点项目，落实好联审联批会议制度，统筹配置生产要素，确保重点项目顺利实施。

（八）深入推进改革开放。按照国家和省部署推进重点领域改革，持续推动体制机制创新，提高对外开放水平。做好重点领域改革工作。继续深化国有企业改革，完善国有资产监管体制，探索国有资产经营、国有资本运作新途径，不断焕发新的发展活力。稳步推进财税改革、事业单位改革以及公益性文化事业单位改革创新。不断深化农村改革，加快集体林权和国有林场改革。继续开展“双改”工作，积极推进户籍制度改革。持续创新要素保障机制。深化银企合作，探索推广新型融资模式。做实做活做强市发展投资公司、城市基础设施投资公司、文化旅游投资公司、新区基础设施投资公司，支持县区投融资平台建设。强化与各商业银行战略合作，争取信贷指标和项目调增额度，实现直接间接融资260亿元。实行最严格的耕地保护制度，完善土地保障机制，强化节约集约用地。提高对外开放水平。重点围绕汽车及零部件、装备制造、电子信息、新材料、生物医药、商贸流通、金融等产业，逐一制定专项招商行动方案，承接引进一批竞争力强、成长性好、关联度高的产业集群项目。借助清明文化节、菊花文化节、第八届中博会等招商引资平台，高起点谋划运作，全方位推介一批重大项目，力争引进省外资金450亿元以上。坚持引资与引智并重，认真落实人才工作配套政策，加强高层次急需紧缺人才引进和管理，实施重大人才工程，用好用活本土人才，加快海内外高层次人才中原创新创业园建设，打造开封人才高地，使人才第一资源为发展第一要务提供强劲支撑。

（九）努力保障和改善民生。继续办好15项民生工程，在学有所教、劳有所得、病有所医、老有所养、住有所居上持续取得新进展，不断提高人民群众幸福指数，努力建设幸福开封。推动实现高质量就业。大力开发中小微企业就业新岗位，努力扩大就业容量，确保新增城镇就业5.8万人、再就业2.3万人，新增农村劳动力转移就业5万人。维护劳动者合法权益，构建和谐劳资关系。持续提高劳动者收入水平，努力实现居民收入与经济发展同步增长，使人民群众共享改革发展成果。切实加强社会保障。继续推动城镇职工社会养老保险与城乡居民养老保险参保扩面，加强城镇职工医保、城镇居民医保、新农合制度建设，提高新农合、城镇居民医保政府补助标准和实际报销水平，推进大病保险和医疗应急救助。进一步完善失业、医疗、工伤、生育保险市级统筹，加快推行社会保障“一卡通”。继续提高企业退休人员基本养老金发放标准和城乡低保、农村五保、优抚对象补助水平。大力发展慈善事业、残疾人事业和老龄事业。完善减灾救灾、社会救助、社会福利制度，建成市社会福利园区，开工建设兰考、通许、开封县等县级福利中心。强力推进保障性安居工程建设。新开工各类保障性住房11200套，其中廉租房1060套、公租房2202套、棚户区改造安置住房7938套，集中力量缓解中低收入群体住房困难问题。加强保障性住房管理，确保工程质量、公平分配和有效入住。努力办好人民满意的教育。始终坚持教育优先发展战略，把立德树人作为教育的根本任务，深化教育领域综合改革，不断加大教育投入，积极鼓励民间资本兴办教育。大力发展学前教育，新改扩建幼儿园200所。推进义务教育均衡发展，抓好中小学布局调整、义务教育学校标准化建设、农村义务教育学校改造，加快集英街中小学、开封高中西校区建设。支持河南大学、黄河水利职业技术学院等高校发展，办好开封大学和开封文化艺术职业学院。大力发展职业教育，持续实施职业教育攻坚计划。提高人民健康水平。加快基层医疗卫生机构综合改革，推进县级公立医院综合改革试点，完善乡镇卫生院、社区卫生服务中心、村卫生室基本药物制度，提高基层卫生服务能力。继续抓好重大疫病防控救治工作，推进市全科医师培训基地、市妇儿医院、市精神病医院等项目建设，为群众提供安全、有效、便捷的医疗卫生服务。广泛开展全民健身运动，办好2013中国郑开国际马拉松赛。加强人口和计划生育服务体系建设，稳定低生育水平。发展文化事业。加快公共文化基础设施建设，深入实施舞台艺术送农民、农村电影放映、广播电视村村通、乡镇文化站、公共电子阅览室、数字图书馆、公共文化设施开放等免费文化惠民工程。加强和创新社会管理。完善社会管理体系，突出城乡社区服务功能，落实村（居）民自治制度，夯实基层社会管理基础。畅通群众诉求表达、利益协调、权益保障渠道，完善信访制度。加强防灾减灾体系建设和应急管理，提高突发事件应对处置能力。加强安全生产和食品药品监管，深化平安开封建设，依法打击违法犯罪活动，确保社会大局和谐稳定，让和谐之风劲吹开封城乡大地。

（十）加强生态文明建设。以绿色发展、循环发展、低碳发展为目标，把生态文明建设融入经济社会发展全过程，努力建设美丽开封。加大环境保护力度，严格环保准入和污染物排放标准，继续强化工程减排、结构减排和管理减排，深入实施环境容量预算管理，探索推进排污权交易。加强PM2.5监测与发布，采取针对性措施改善空气质量。完成黄汴河、东护城河截污工程，加大贾鲁河、惠济河、涡河故道等重点河流治理力度。加快污水处理厂建设进度，建成生活垃圾焚烧发电项目，强化饮用水源地和湿地保护。加强农村环境连片综合整治，切实控制面源污染。启动生态市创建工作，加快生态村镇建设步伐，完成郑民高速和大广高速两侧绿化。深入推进重点领域节能降耗，加快实施节能改造、节能惠民、节能技术产业化示范推广重点工程，降低资源消耗强度，增强可持续发展后劲。

继续加强国防动员和后备力量建设，支持驻汴解放军、武警部队、预备役部队、民兵和人民防空建设，深入开展双拥共建，巩固军政军民团结，促进军民融合式发展。支持工会、共青团、妇联等人民团体发挥桥梁纽带作用，注重发挥工商联等社会团体作用。全面贯彻党的民族宗教政策，依法管理民族宗教事务。继续做好外事、侨务、对台、统计、史志、气象、地震、社科研究等工作。

三、转变工作作风，提高行政能力

做好今年的政府工作，必须把转变职能、改进作风、提高能力、提升效能作为今后一个阶段的关键，严格落实中央关于改进工作作风、密切联系群众“八项规定”，切实转变领导方式和工作方式，深入推进“两转两提”，优化经济发展环境，提高政府执行力和公信力。

（一）强化服务理念。加强公务员队伍教育和管理，牢固树立全心全意为人民服务的宗旨意识，忠实履行服务群众、改善民生职责，建设人民满意的服务型政府。立足实际，加强学习，提高服务大局的能力、破解难题的能力、创新发展的能力、处理复杂问题的能力和推动科学发展的能力。坚持问政于民、问需于民、问计于民，使工作决策和工作推进建立在深厚的民意基础之上。

（二）提高行政效能。扎实推进效能建设年，下决心治庸治懒治散治贪，不断提高行政效能和服务质量。深化行政管理体制改革，转变政府职能和管理方式，建立规范高效的行政管理运行机制。推进行政审批制度改革，精简审批事项，减少审批环节，规范审批行为，推行行政审批事项交办制度，提高审批效率。认真落实政务公开、服务承诺、限时办结等制度，提高办事效率。完善目标责任制、工作评价机制，推行绩效管理，强化效能问责，严格责任追究，确保各级公务人员忠诚履职，勤勉敬业。

（三）转变工作作风。大力弘扬焦裕禄精神，坚持出实招、办实事、求实效，深入实际，深入基层，深入群众，扑下身子抓落实，集中精力解难题。大力开展调查研究，重点围绕市委、市政府确定的十个重大课题，学先进、找差距、谋大事、求大变。发扬艰苦奋斗精神，严格控制和压缩行政经费。精简会议活动、文件简报，减少检查评比、迎来送往，严禁铺张浪费。认真落实党风廉政建设责任制，强化反腐倡廉，完善惩防体系，深入开展政风行风建设，规范权力运行，建设人民满意、人民支持的清廉政府。

（四）推进依法行政。严格按照法定权限和程序行使权力、履行职责。坚持科学民主依法决策，完善公众参与、专家论证和政府决策相结合的决策机制。加强行政行为的规范和监督，确保严格执法、公正执法、文明执法。严格执行人大及其常委会的决议决定，自觉接受法律监督和工作监督，主动接受人民政协民主监督，支持人民政协履行职能。认真办理人大议案、代表建议和政协提案，主动听取各民主党派、工商联、各人民团体和人民群众的意见建议。虚心接受社会监督和舆论监督，让人民更加有效地监督政府，使政府更好地服务人民，更好地为人民办事，更好地为人民谋福祉。

各位代表!

党的十八大提出了全面建成小康社会的目标，中原经济区建设和“五大攻坚战”开启了开封跨越发展的新征程。面对加快发展新形势，面对中原经济区建设新机遇，面对全市人民新期待，我们深感任务艰巨、责任重大、使命光荣！让我们高举中国特色社会主义伟大旗帜，紧密团结在以习近平同志为总书记的党中央周围，在省委、省政府和市委的坚强领导下，在市人大、市政协的监督支持下，振奋精神，持续求快，务实发展，努力创造经得起实践、人民和历史检验的工作业绩，奋力开创开封快速崛起的新局面！
